# Supplementary material for: Genome-scale phylogenetic analyses confirm Olpidium as the closest living zoosporic fungus to the non-flagellated, terrestrial fungi
Source: Sci Rep. 2021 Feb 5;11:3217. doi: 10.1038/s41598-021-82607-4 (PMC7865070; doi:10.1038/s41598-021-82607-4)
Supplement: Supplementary file 1 — Supplementary Information. [file 41598_2021_82607_MOESM1_ESM.pdf]

Genome-scale phylogenetic analyses confirm *Olpidium* as the closest living zoosporic fungus to the non-flagellated, terrestrial fungi

Ying Chang[1], D'Ann Rochon[2], Satoshi Sekimoto[3,4], Yan Wang[5,6,7,8], Mansi Chovatia[9], Laura Sandor[9], Asaf Salamov[9], Igor V. Grigoriev[9,10], Jason E. Stajich[5,6], Joseph W. Spatafora[1]

*Correspondence to changyi@oregonstate.edu

Fig. S1. The flowchart showing the procedure and results of phylogenetic reconstruction, topology tests and polytomy tests.

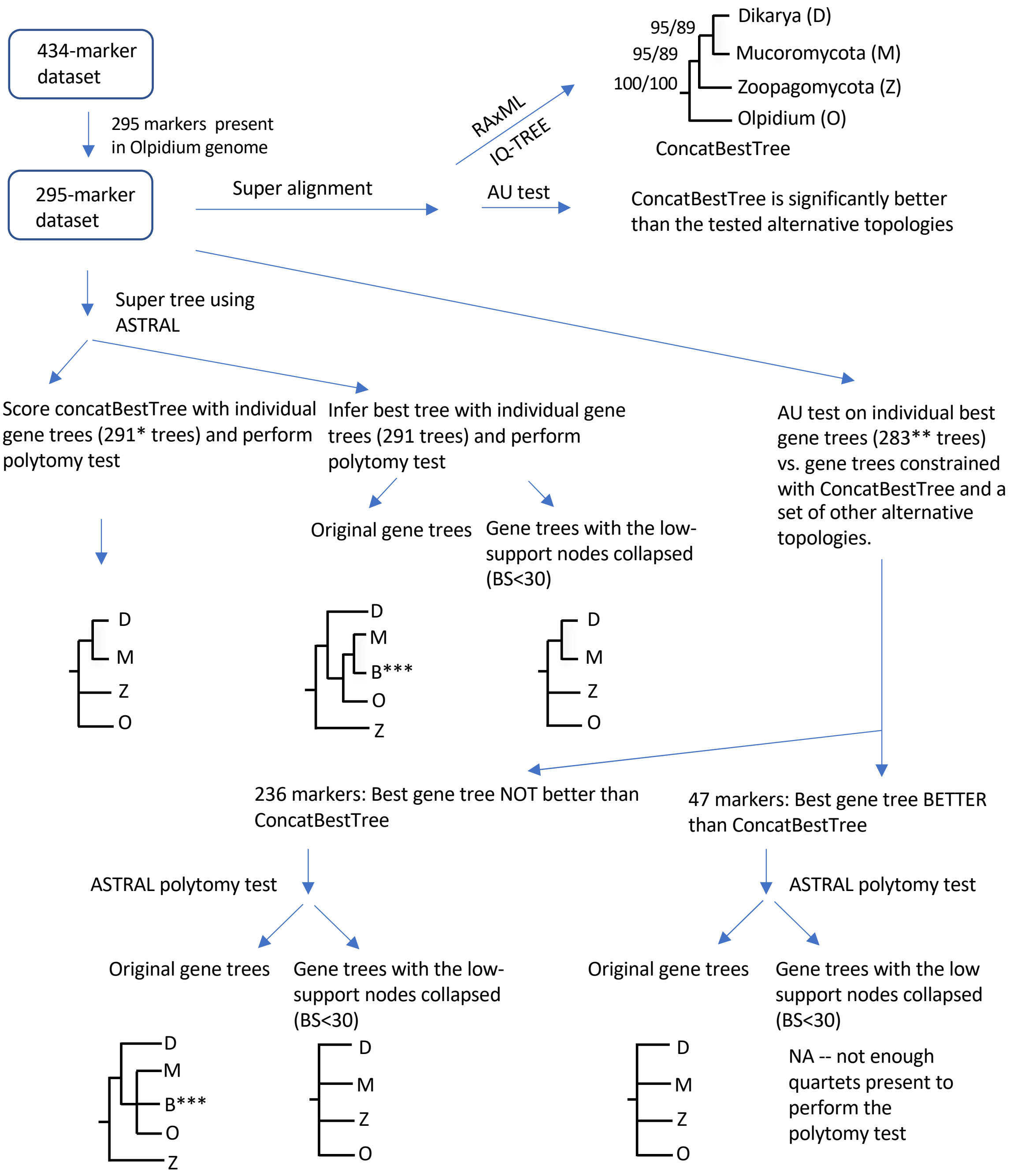

*: Four individual marker alignments contain identical sequences from two different species or contain sequence(s) with only undetermined values; hence no RAxML analysis on those markers.

**: No constrained-tree search is done for four markers due to the large number of missing species for those markers. Together with the four markers with all-undetermined sequences/identical sequences for two different species, a total of eight markers are not eligible for AU test.

***: B stands for *Basidiobolus*.

Table S1. Genome information of the 113 taxa included in the phylogenetic reconstruction.

Species	GenBank accession No./JGI Web Portal (reference)
Allomyces macrogynus ATCC 38327	ACDU00000000.1
Anaeromyces robustus	https://genome.jgi.doe.gov/Anasp1/ [1]
Arabidospsis thalina	GCA_000001735.2 [2]
Arthrobotrys oligospora ATCC 24927	ADOT00000000 [3]
Aspergilus fumigaus ATCC 1015	ACJE00000000 [4]
Atractiellales sp.	https://mycocosm.jgi.doe.gov/Atrsp2/
Auricularia subglabra	https://mycocosm.jgi.doe.gov/Aurde3_1/A [5]
Backusella circina FSU 941	http://genome.jgi.doe.gov/Bacci1
Basidiobolus heterosporus B8920	GCA_000697455.1 [6]
Basidiobolus meristosporis B9252	GCA_000697375.1 [7]
Basidiobolus meristosporis CBS 931.73	https://mycocosm.jgi.doe.gov/Basme2finSC/ [7]
Batrachochytrium dendrobatidis JAM81	ADAR00000000.1
Bifiguratus adelaidae AZ0501	MVBO00000000 [8]
Blakeslea trispora NRRL 2456	https://mycocosm.jgi.doe.gov/Blatri1/
Blastobotrys adeninivorans LS3	https://mycocosm.jgi.doe.gov/Arxad1/ [9]
Botrytis cinerea B05.10	GCA_00143535.4 [10]
Calcarisporiella thermophila CBS 279.70	https://gb.fungalgenomics.ca/portal/
Capniomyces stellatus MIS-10-108	LUVW010000000 [11]
Capsaspora owczarzaki ATCC 30864	ACFS00000000.2 [12]
Catenaria anguillulae PL171	http://genome.jgi.doe.gov/Catan1 [7]
Cenococcum geophilum 1.58	https://mycocosm.jgi.doe.gov/Cenge3/ [13]
Cephaloascus albidus ATCC 66658	https://mycocosm.jgi.doe.gov/Cepfr1_1
Chlamydomonas reinhardtii CC-503	ABCN00000000.2 [14]
Chytriomyces sp. MP 71	https://mycocosm.jgi.doe.gov/Chytri1/
Clavispora lusitaniae ATCC 42720	AAFT00000000.1 [15]
Clavulina sp. PMI 390	https://mycocosm.jgi.doe.gov/ClaPMI390
Coemansia reversa NRRL 1564	JZJC00000000 [16]
Cokeromyces recurvatus B5483	https://mycocosm.jgi.doe.gov/Cokrec1/
Conidiobolus coronatus NRRL 28638	JXYT00000000 [16]
Conidiobolus incongruous B7586	GCA_000697335.1 [16]
Conidiobolus thromboides FSU 785	http://genome.jgi.doe.gov/Conth1
Coprinopsis cinerea Okayama7_130	AACS00000000.2 [17]
Cryptococcus neoformans JEC21	GCA_000149245.3 [18]
Cunninghamella echinulata NRRL 1832	https://mycocosm.jgi.doe.gov/Cunech1/
Cyberlindnera jadinii NRRL Y-1542	https://mycocosm.jgi.doe.gov/Cybja1/ [19]
Dacryopinax sp. DJM-731	AEUS00000000.1 [5]
Dictyostelium discoideum	AAFI00000000.2 [20]
Dimargaris cristalligena RSA 468	https://mycocosm.jgi.doe.gov/DimcrSC1/ [21]
Drosophila melanogaster vr6.04	http://flybase.org [22]
Encephalitozoon intestinalis ATCC 50506	GCA_000146465.1 [23]
Endogone sp. FLAS F-59071	RBNK00000000.1 [24]

Species	GenBank accession No./JGI Web Portal (reference)
Entophlyctis helioformis JEL 805	https://mycocosm.jgi.doe.gov/Enthel1/
Exobasidium vaccinia MPITM	PRJNA196015
Funneliformis mosseae DAOM-236685*	https://github.com/zygolife/AMF_Phylogenomics [25]
Ganoderma sp. 10597 SS1	https://mycocosm.jgi.doe.gov/Gansp1/ [26]
Gloeophyllum trabeum ATCC 11539	GCA_000344685.1 [5]
Gonapodya prolifera JEL478	LSZK00000000 (Chang et al. 2015) [16]
Hesseltinella vesiculosa NRRL 3301	http://genome.jgi.doe.gov/Hesve2finisherSC [7]
Homoloaphlyctis polyrhiza JEL142	AFSM01000000.1 [27]
Jimgerdemmannia flammicorona AD002	https://mycocosm.jgi.doe.gov/Jimfl_AD_1/ [24]
Jimgerdemmannia flammicorona GMNB39	https://mycocosm.jgi.doe.gov/Jimfl_GMNB39_1/ [24]
Jimgerdemmannia lactiflua OSC 162217	https://mycocosm.jgi.doe.gov/Jimlac1/ [24]
Lichtheimia corymbifera FSU 9682	CBTN000000000.1 [28]
Lichtheimia hyalospora FSU 10163	http://genome.jgi.doe.gov/Lichy1
Linderina pennispora ATCC 12442	http://genome.jgi.doe.gov/Linpe1 [7]
Martensiomyces pterosporus CBS 209.56	http://genome.jgi.doe.gov/Marpt1
Monosiga brevicolis MX1	ABFJ00000000.1 [29]
Mortierella elongata AG-77	http://genome.jgi.doe.gov/Morel2 [30]
Mortierella verticillata NRRL 6337	AEVJ00000000.1
Mucor circinelloides CBS 277.49	http://genome.jgi.doe.gov/Mucci2 [31]
Neurospora crassa OR74A	AABX00000000.3 [32]
Olpidium bornovanus S191	JAEFCI010000000
Orpinomyces sp. C1A	ASRE00000000.1 [33]
*Pandora formicae**	GCRV00000000.1 [34]
Paraglomus brasilianum DAOM-240472*	https://github.com/zygolife/AMF_Phylogenomics [25]
Phycomyces blakesleeanus NRRL 1555	http://genome.jgi.doe.gov/Phybl2 [35]
Piptocephalis cylindrospora RSA 2659	http://genome.jgi.doe.gov/Pipcy2/Pipcy2.home.html [21]
Piromyces sp. E2	http://genome.jgi.doe.gov/PirE2_1 [1]
Puccinia graminis f. sp. tritici CRL 75-36-700-3	AAWC0000000.1 [36]
Racocetra castanea BEG-1*	https://github.com/zygolife/AMF_Phylogenomics [25]
Ramicandelaber brevisporus CBS 109374	http://genome.jgi.doe.gov/Rambr1
Rhizophagus diaphanous MUCL 43196	http://genome.jgi.doe.gov/Rhidi1 (Morrin *et al.*, 2019)
Rhizophagus irregularis DAOM 181602	JARB00000000.1 [38]
Rhizopus delemar RA99-880	AACW0000000.2 [39]
Rhizopus microsporus var chinesis CCTCCM201021	CCYT00000000.1 [40]
Rhizopus microsporus var microsporus ATCC 52813	http://genome.jgi.doe.gov/Rhimi1_1 [7]
Rozella allomycis CSF55	ATJD00000000.1 [41]
Saccharomyces serevisiae S288C.vR642-1	http://yeastgenome.org/ [42]
Saksenaea vasiformis B4078	JNDT00000000.1 [6]
Schizosaccharomyces pombe 972h-.vASM294	http://www.pombase.org/ [43]
Scutellospora calospora INVAM-IL209*	https://github.com/zygolife/AMF_Phylogenomics [25]
Spizellomyces punctatus DAOM BR117	ACOE00000000.1 [44]
Umbelopsis ramanniana NRRL 5844	http://genome.jgi.doe.gov/Umbra1
Ustilago maydis 521 v190413	AACP00000000.2 [45]

Species	GenBank accession No./JGI Web Portal (reference)
Yarrowia lipolytica CLIB 122	GCA_000002525.1 [46]
Zoophthora radicans ATCC 208865	http://genome.jgi.doe.gov/ZooradStandDraft_FD/

* Taxa with only transcriptome data.

References:

1. Haitjema, C. H. *et al.* A parts list for fungal cellulosomes revealed by comparative genomics. *Nat. Microbiol.* **2**, 17087 (2017).
2. Theologis, A. *et al.* Sequence and analysis of chromosome 1 of the plant Arabidopsis thaliana. *Nature* **408**, 816–820 (2000).
3. Yang, J. *et al.* Genomic and Proteomic Analyses of the Fungus Arthrobotrys oligospora Provide Insights into Nematode-Trap Formation. *PLoS Pathog.* **7**, e1002179 (2011).
4. Andersen, M. R. *et al.* Comparative genomics of citric-acid-producing Aspergillus niger ATCC 1015 versus enzyme-producing CBS 513.88. *Genome Res.* **21**, 885–97 (2011).
5. Floudas, D. *et al.* The Paleozoic origin of enzymatic lignin decomposition reconstructed from 31 fungal genomes. *Science (80-.).* **336**, 1715–1719 (2012).
6. Chibucos, M. C. *et al.* An integrated genomic and transcriptomic survey of mucormycosis-causing fungi. *Nat. Commun.* **7**, 12218 (2016).
7. Mondo, S. J. *et al.* Widespread adenine N6-methylation of active genes in fungi. *Nat. Genet.* **49**, 964–968 (2017).
8. Torres-Cruz, T. J. *et al.* Bifiguratus adelaidae, gen. et sp. nov., a new member of Mucoromycotina in endophytic and soil-dwelling habitats. *Mycologia* **109**, 363–378 (2017).
9. Kunze, G. *et al.* The complete genome of Blastobotrys (Arxula) adeninivorans LS3 - a yeast of biotechnological interest. *Biotechnol. Biofuels* **7**, 66 (2014).
10. Staats, M. & van Kan, J. A. L. Genome update of Botrytis cinerea strains B05.10 and T4. *Eukaryot. Cell* **11**, 1413–1414 (2012).
11. Wang, Y., White, M. M. & Moncalvo, J.-M. Draft Genome Sequence of Capniomyces stellatus, the Obligate Gut Fungal Symbiont of Stonefly. *Genome Announc* **4**, 804–820 (2016).
12. Suga, H. *et al.* The Capsaspora genome reveals a complex unicellular prehistory of animals. *Nat Commun* **4**, 2325 (2013).
13. Peter, M. *et al.* Ectomycorrhizal ecology is imprinted in the genome of the dominant symbiotic fungus Cenococcum geophilum. *Nat Commun* **7**, 12662 (2016).
14. Merchant, S. S. *et al.* The Chlamydomonas Genome Reveals the Evolution of Key Animal and Plant Functions. *Science (80-.).* **318**, 245–250 (2007).
15. Butler, G. *et al.* Evolution of pathogenicity and sexual reproduction in eight Candida genomes. *Nature* **459**, 657–662 (2009).
16. Chang, Y. *et al.* Phylogenomic Analyses Indicate that Early Fungi Evolved Digesting Cell Walls of Algal Ancestors of Land Plants. *Genome Biol. Evol.* **7**, 1590–1601 (2015).
17. Stajich, J. E. *et al.* Insights into evolution of multicellular fungi from the assembled chromosomes of the mushroom Coprinopsis cinerea (Coprinus cinereus). *Proc. Natl. Acad. Sci. U. S. A.* **107**, 11889–94 (2010).
18. Loftus, B. J. *et al.* The genome of the basidiomycetous yeast and human pathogen Cryptococcus neoformans. *Science* **307**, 1321–4 (2005).
19. Riley, R. *et al.* Comparative genomics of biotechnologically important yeasts. *Proc. Natl. Acad. Sci.* **113**, 9882–9887 (2016).
20. Eichinger, L. *et al.* The genome of the social amoeba Dictyostelium discoideum. *Nature* **435**, 43–57 (2005).
21. Ahrendt, S. R. *et al.* Leveraging single-cell genomics to expand the fungal tree of life. *Nat. Microbiol.* **3**, 1417–1428 (2018).
22. Adams, M. D. *et al.* The genome sequence of Drosophila melanogaster. *Science (80-.).* **287**, (2000).
23. Corradi, N., Pombert, J.-F., Farinelli, L., Didier, E. S. & Keeling, P. J. The complete sequence of the smallest known nuclear genome from the microsporidian Encephalitozoon intestinalis. *Nat. Commun.* **1**, 77 (2010).
24. Chang, Y. *et al.* Phylogenomics of Endogonaceae and evolution of mycorrhizas within Mucoromycota. *New Phytol.* **222**, 511–525 (2019).
25. Beaudet, D. *et al.* Ultra-low input transcriptomics reveal the spore functional content and phylogenetic affiliations of poorly studied arbuscular mycorrhizal fungi. *DNA Res.* dsx051–dsx051 (2017). doi:10.1093/dnares/dsx051
26. Binder, M. *et al.* Phylogenetic and phylogenomic overview of the Polyporales. *Mycologia* **105**, 1350–73 (2013).
27. Joneson, S., Stajich, J. E., Shiu, S.-H. & Rosenblum, E. B. Genomic transition to pathogenicity in chytrid fungi. *PLoS Pathog.* **7**, e1002338 (2011).
28. VU, S. *et al.* Gene Expansion Shapes Genome Architecture in the Human Pathogen Lichtheimia Corymbifera: An Evolutionary Genomics Analysis in the Ancient Terrestrial Mucorales (Mucoromycotina). *PLoS Genet.* **10**, (2014).
29. King, N. *et al.* The genome of the choanoflagellate Monosiga brevicollis and the origin of metazoans. *Nature* **451**, 783–8 (2008).
30. Uehling, J. *et al.* Comparative genomics of *Mortierella elongata* and its bacterial endosymbiont *Mycoavidus cysteinexigens*.

Environ. Microbiol. **19**, 2964–2983 (2017).
31. Navarro-Mendoza, M. I. *et al.* Early Diverging Fungus Mucor circinelloides Lacks Centromeric Histone CENP-A and Displays a Mosaic of Point and Regional Centromeres. *Curr. Biol.* **29**, 3791-3802.e6 (2019).
32. Galagan, J. E. *et al.* The genome sequence of the filamentous fungus Neurosporacrassa. *Nature* **422**, 859–868 (2003).
33. Youssef, N. H. *et al.* The genome of the anaerobic fungus Orpinomyces sp. strain C1A reveals the unique evolutionary history of a remarkable plant biomass degrader. *Appl. Environ. Microbiol.* **79**, 4620–34 (2013).
34. Małagocka, J., Grell, M. N., Lange, L., Eilenberg, J. & Jensen, A. B. Transcriptome of an entomophthoralean fungus (Pandora formicae) shows molecular machinery adjusted for successful host exploitation and transmission. *J. Invertebr. Pathol.* **128**, 47–56 (2015).
35. Corrochano, L. M. *et al.* Expansion of Signal Transduction Pathways in Fungi by Extensive Genome Duplication. (2016). doi:10.1016/j.cub.2016.04.038
36. Duplessis, S. *et al.* Obligate biotrophy features unraveled by the genomic analysis of rust fungi. *Proc Natl Acad Sci U S A* **108**, (2011).
37. E, M. *et al.* Comparative Genomics of Rhizophagus Irregularis, R. Cerebriforme, R. Diaphanus and Gigaspora Rosea Highlights Specific Genetic Features in Glomeromycotina. *New Phytol.* **222**, (2019).
38. Tisserant, E. *et al.* Genome of an arbuscular mycorrhizal fungus provides insight into the oldest plant symbiosis. *Proc. Natl. Acad. Sci. USA* **110**, 20117–20122 (2013).
39. Ma, L. J. *et al.* Genomic analysis of the basal lineage fungus Rhizopus oryzae reveals a whole-genome duplication. *PLoS Genet* **5**, e1000549 (2009).
40. Wang, D., Wu, R., Xu, Y. & Li, M. Draft Genome Sequence of Rhizopus Chinensis CCTCCM201021, Used for Brewing Traditional Chinese Alcoholic Beverages. *Genome Announc.* **1**, (2013).
41. James, T. Y. *et al.* Shared Signatures of Parasitism and Phylogenomics Unite Cryptomycota and Microsporidia. *Curr. Biol.* **23**, 1548–1553 (2013).
42. Goffeau, A. *et al.* Life with 6000 Genes. *Science (80-.).* **274**, 546–567 (1996).
43. Wood, V. *et al.* The Genome Sequence of Schizosaccharomyces Pombe. *Nature* **415**, (2002).
44. Russ, C. *et al.* Genome Sequence of Spizellomyces punctatus. *Genome Announc.* **4**, (2016).
45. Kamper, J. *et al.* Insights from the genome of the biotrophic fungal plant pathogen Ustilago maydis. *Nature* **444**, 97–101 (2006).
46. Dujon, B. *et al.* Genome Evolution in Yeasts. *Nature* **430**, (2004).

Table S2. Summary of branch support regarding the placement of *Olpidium* and the main non-flagellated terrestrial fungal groups. All the ML-based analyses (i.e., RAxML and IQ-TREE analyses) recovered the same branching patterns in their best tree, (O, (Z, (M, D))), while (((O, (B, M)), D), Z') and ((B,(M, D)), Z') were the optimal topologies inferred by the ASTRAL analyses based on original gene trees and on gene trees with weak branches collapsed (BS < 30), respectively. Bootstrap higher than 50 and posterior probabilities higher than 0.5 are shown. B – *Basidiobolus*; D – Dikarya; M – Mucoromycota; O – *Olpidium bornavanus;* Z – Zoopagomycota; Z' -- Zoopagomycota without *Basidiobolus*.

Analysis	RAxML		IQ-TREE		ASTRAL	
--no. markers	295	295	295	295	295	[1]295
--models	LG+G	LG+G	LG+F+R10	LG+F+R10*H4	[2]LG+G	[2]LG+G
--other settings	partitioned	Non-partitioned	non-partitioned	non-partitioned	[3]original trees	[4]BS<30 collapsed
(O, Z, M, D)	100	100	100	100	100	1
(Z, M, D)	66	89	96	95	--	--
(M, D)	68	89	96	95	--	--
(O, M, B)	--	--	--	--	82	--
(M, B)	--	--	--	--	83	--
(O, M, D)	--	--	--	--	--	0.85

[1]Branch support shown is local posterior probabilities, computed based on a transformation of the percentage of quartets in individual gene trees that agree or disagrees with a branch.
[2]LG+G was the substitution model used in the inference of individual gene trees using RAxML.
[3]The original best individual gene trees were used in the ASTRAL analysis.
[4]In this ASTRAL analysis, the weakly supported branches (bootstrap < 30) were collapsed for each best individual gene tree.

Table S3. Summary of branch support regarding the placement of *Olpidium* and the main non-flagellated terrestrial fungal groups in RAxML analyses with faster-evolving sites removed. The amino acid sites in the 295-marker concatenated matrix were sorted to ten rate categories, with category 10 being the fastest evolving sites. We sequentially removed the fast-evolving sites and perform RAxML analysis with 100 bootstrap replicates and PROTGAMMALG model. The arrangement of (O, (Z, (M, D))) was recovered in all the RAxML analyses. Bootstrap higher than 50 and posterior probabilities higher than 0.5 are shown. D – Dikarya; M – Mucoromycota; O – *Olpidium bornavanus;* Z – Zoopagomycota.

TIGER rate category removed	None	10	9,10	8,9,10	7,8,9,10	6,7,8,9,10	5,6,7,8,9,10
(O, Z, M, D)	100	100	100	100	100	100	100
(Z, M, D)	89	72	82	77	77	70	82
(M, D)	89	72	85	82	79	74	82

Table S4. Fossil calibrations and gene family GH28 expansion calibration used in MCMCTREE analyses.

**Clade**	**Fossil**	**Calibration Age**	**Calibration Constraint Setting**	**Ref.**
Blastocladiomycota	*Palaeoblastocladia milleri*	407	lower minimum bound	1
Chytridiomycota	*Krispiromyces discoides*	407	lower minimum bound	1
Endogonaceae	*Jimwhitea circumtecta*	247	lower minimum bound	1
Mucorales	*Protoascon missouriensis*	315	lower minimum bound	1
Ascomycota	*Paleopyrenomycites devonicus*	407	lower minimum bound	1
Basidiomycota	Clamp connections	330	lower minimum bound	1
Chytridiomycota + *Olpidium* + Zoopagomycota + Mucoromycota + Dikarya	GH28 expansion in Fungi	1100	upper maximum bound	2

1) Taylor TN, Krings M, Taylor EL. 2015. Fossil Fungi. Academic Press, London, 382 pp.

2) Chang, Ying et al. 2015. “Phylogenomic Analyses Indicate That Early Fungi Evolved Digesting Cell Walls of Algal Ancestors of Land Plants.” *Genome Biology and Evolution* 7(6):1590–1601.
